# Effects of storage on brown rice (*Oryza sativa* L.) metabolites, analyzed using gas chromatography and mass spectrometry

**DOI:** 10.1002/fsn3.1589

**Published:** 2020-05-18

**Authors:** Changyuan Wang, Yuchao Feng, Shu Zhang, Tianxin Fu, Yanan Sheng, Yiwei Zhang, Yingjun Jiang, Miao Yu, Liyuan Zhang, Dongjie Zhang

**Affiliations:** ^1^ College of Food Heilongjiang Bayi Agricultural University Daqing China; ^2^ Daqing Center of Inspection and Testing for Agricultural Products Ministry of Agriculture Daqing China

**Keywords:** brown rice, gas chromatography–mass spectrometry, metabolism, storage

## Abstract

Metabolomic studies were carried out using gas chromatography and mass spectrometry (GC‐MS) on Daohuaxiang variety rice (*Oryza sativa* L.) from the Wuchang Geographical Indication Rice Protection Area in Heilongjiang Province, to investigate the effects of storage on brown rice metabolism. The data were subjected to principal component analysis (PCA), orthogonal partial least squares‐discriminant analysis (OPLS‐DA), and cluster analysis using software such as SIMCA. Analysis of the samples led to the identification of a total of 160 metabolites. No significant differences were found in the amount of metabolites before and after storage. A total of 31 differential metabolites were screened, and the changes in metabolite content showed a “reverse change” overall. Storage significantly changed the content of various metabolites in rice, with fatty acids impacted most significantly. Metabolic pathway analysis revealed that fatty acid biosynthesis is a key metabolic pathway in rice storage. The degradation of brown rice quality caused by storage is closely related to the composition and content of its metabolites, and that change in lipid content significantly affects brown rice quality during storage.

## Graphical abstract

Storage has a small effect on the amount of brown rice metabolites and a large effect on the composition of metabolites. Storage has a significant effect on the content of lipids and carbohydrates, and has a “reverse change” trend. Storage has the greatest impact on fatty acid synthesis pathways. Lauric acid is a key metabolite for lipid metabolism during storage of Daohuaxiang variety rice. Lipids are the main factors affecting brown rice quality during storage.

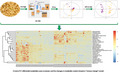

## 1 INTRODUCTION

Metabolomics is the study of the endogenous metabolites in organisms and their changes. It is a science utilizing qualitative and quantitative analysis of all low molecular weight (<1,000) metabolites of a biological cell or organism during a specific physiological period (Tang & Wang, 2007). Since its introduction in the 1980s (Sauter, Lauer, & Fritsch, 1991), metabolomics has been cited as an analytical method employed in over 100 research publications on plant metabolites, which were different from phytochemicals (Zhang, Yu, Wang, & Zhang, 2019). Rice (*Oryza sativa* L.) is the primary food crop in China (Liu et al., 2013; Wang, Feng, Sun, Cong, & Zhu, 2013), in 2018, China's rice output reached 212 million tons, while consumption reached 193 million tons. Wuchang rice output reached about 1 million tons, which is in short supply in the market. Storage is an important part of rice harvesting and distribution. Brown rice is susceptible to microbial contamination and oxidative deterioration during storage, resulting in aging‐related phenomena such as reduced physiological activity, fat oxidation, and protein degradation (Chen, Hung, & Lin, 2015; Xu, Cheng, Cai, & Wang, 2005). The metabolic activity of brown rice during the storage period may lead to a deterioration in its quality. Currently, research on brown rice storage mostly focuses on changes in lipids, amino acids, starch, protein, and enzymes in brown rice, and microorganisms during the aging process (Bolling, Hampel, & Elbaya, 1977; Chrastil & Zarins, 1992; Jin, Zhu, Liu, Zhao, & Yuan, 2017; Li, Wu, Wu, Lin, & Liang, 2013; Shao et al., 2009; Shin, 1991; Yang, Jiang, Fu, Jiang, & Li, 2018) because these factors significantly impact brown rice quality. It is feasible to analyze the changes in various metabolites during the process of brown rice aging. Studies have also indicated that the growth environment affects the metabolism and composition of crops (Choudury & Juliano, 1980; Liu & Cheng, 2006). In the present study, metabolomics based on gas chromatography–mass spectrometry (GC‐MS) (Duan & Qi, 2015) was used for the isolation and identification of metabolites from Daohuaxiang variety brown rice in Wuchang Geographical Indication Rice Protection Area in Heilongjiang Province. Metabolomics was used to study the effects of storage on brown rice metabolism. The process of brown rice quality change was analyzed from the perspective of metabolomics, the changes in brown rice quality were visualized, and the mechanism of brown rice quality deterioration was determined.

## 2 MATERIALS AND METHODS

### 2.1 Materials and reagents

#### 2.1.1 Plant material

Daohuaxiang variety rice (*Oryza sativa* L.) samples were collected from Wuchang City, Heilongjiang Province, China (abbreviated as WC). Heilongjiang Province is China's largest grain producer. Wuchang City is located in the southernmost part of Heilongjiang Province. It belongs to the second and third accumulated temperate zones, with a continental monsoon climate in the mid‐temperate zone, between 44°4′ to 45°26′N and 126°33′ to 128°14′E. The soil humus layer is thick, with high basic fertility, and the field water‐holding capacity is between 20% and 40%. These factors make it the most suitable area for japonica brown rice cultivation in China. Daohuaxiang variety rice from Wuchang origin is a product of Chinese geographical indication. It is named “Daohuaxiang variety rice” because of its aroma of leaves and rice flowers. It is a unique variety of long‐grain, fragrant japonica, particularly suitable for planting in the Wuchang area. Influenced by the good geography and climate of the production area, it has greater dry matter accumulation, moderate amylose content, and higher amylopectin content compared to other varieties. Due to the large temperature difference between day and night in the region, more soluble double‐chain sugars accumulate in the rice. The resulting rice grains are full, hard in texture, and clear in color. Wuchang rice is light, slightly sweet, soft, slightly sticky, aromatic, and refreshing. The surface of the rice grains is shiny. The sample was divided into two groups. The first group (the control) was collected in 2015 (Abbreviation: 15WC), and the second group was the 2015 sample after storage for 1 year (Abbreviation: ZCWC). Sampling points were set according to the protection scope of the Wuchang Geographical Indication Rice Protection Area and the size of the planting area. Samples were collected from nine towns and villages in the Wuchang area during the rice maturity period. They were collected according to the five‐point sampling method and the representative sampling principle (Bao, 2018). Each plot was randomly set with 5 repeat points. The main planting variety in the protected area, “Daohuaxiang variety rice,” was selected randomly, and 1–2 kg of rice ears was collected at each sampling point. The collected samples were washed to remove impurities and air‐dried to a moisture content of <14%. The grains from the top of the rice ears were threshed and packed into nylon net bags to a weight of 200 g per bag. The bags were turned once every 10 days to check for mildew on the rice. The bags were stored in a cool and ventilated storage room at the temperature of approximately 18°C for 1 year. A hulling machine was used to hull rice prior to analysis, and the experimental sample was the hulled grain of brown rice, with intact bran.

#### 2.1.2 Chemicals, materials, and apparatus

Methanol and pyridine (≥99.0% purity) (chromatographic grade) were purchased from ALADDIN Reagent Co., Ltd. Methoxyamine hydrochloride (98.5% purity) and N, O‐bis (trimethylsilyl) trifluoroacetamide (99% BSTFA + 1% TMCS) were purchased from Macklin Reagent Co. Ltd. HPLC‐grade water was obtained from a Milli‐Q Water Purification System (Millipore Corp.) and used to prepare all aqueous solutions. All other reagents of analytical grade were purchased from Beijing Chemical Factory. The 7890A/5975C GC‐MS (Agilent J&W Scientific) equipment was used. Chromatographic separation of metabolites was performed on an HP‐5ms (30 m × 0.25 mm × 0.25 μm) (Agilent J&W Scientific). The Termovap Sample Concentrator (Automatic Science Instrument Co., Ltd), DHG‐9123A electric heating drying oven (Jinghong Laboratory Equipment Co., Ltd.), an FC2K Rice Huller (Dazhu Production Co., Ltd.), Incubator (Senxin Instrument Co., Ltd.), and TGL‐16B High‐Speed Centrifuge (Anting Instrument Co., Ltd.) were used during the experimental procedures.

### 2.2 Sample preparation

The sample processing method and chromatographic method were carried out according to those described in the literature (Cheng et al., 2016; Zhou et al., 2012; Zhang, Yu, Wang, & Zhang, 2019) with slight modifications. Before sampling, mix the brown rice stored in the nylon bag thoroughly. Weigh 50 g of rice and shell it with a sheller, and 5 g of brown rice samples was ground with liquid nitrogen. After grinding, the brown rice flour was passed through a 100‐mesh sieve. In a 1.5‐ml EP tube, 800 µl of methanol solution was added to 50 mg sample and vortexed for 30 s. Subsequently, they were centrifuged at 17 400 *g* for 15 min at 4°C. A volume of 200 μl supernatant was transferred to sample vial and dried with nitrogen. Derivatization: 30 μl of pyridine hydrochloride solution was added to the concentrated sample and mixed well until it was completely dissolved. The sample solution was incubated at 37°C for 90 min, and then, 30 μl of BSTFA was added. The mixture was placed in an oven at 70°C for 60 min and then taken for testing.

### 2.3 GC‐MS analysis

Analytical platform: Agilent 7890A/5975C GC‐MS. A 1 µl sample was injected with the autosampler. Gas chromatography analysis was performed on a 30 m HP‐5ms column with 0.25 mm inner diameter and 0.25 mm film thickness (Agilent J&W Scientific). The instrument parameters were 280°C inlet temperature, 230°C EI ion source temperature, and 150°C quadrupole temperature. High‐purity helium (purity >99.999%) was used as the carrier gas. The sample was injected without splitting, and the injection volume was 1.0 μl. The heating procedure was initiated at an initial temperature of 80°C for 2 min. Temperature was then raised to 320°C at rate of 10°C/min for 6 min. Mass spectrometry was performed using the full scan mode. The mass spectrometry range was 50–550 (m/z). The detection time was 37 min. To ensure the accuracy of the experimental results, the samples of 2015 and stored samples of 1 year were tested at the same time and under same conditions. Fresh brown rice samples were quenched with liquid nitrogen and stored at −80°C to terminate the metabolic activity, and then were analyzed together with the stored samples of 1 year. To ensure the stability and accuracy of instrument, a QC sample was tested after analyzing of 10 samples intervals.

### 2.4 Data analysis

Gas chromatography and mass spectrometry data analysis was performed at Shanghai Biotree Biotech Co. Ltd. The GC‐MS data were extracted and preprocessed using the XCMS software package under the R software platform, and the edited data matrix was imported into SIMCA software (V14.1, Sartorius Stedim Data Analytics AB) for principal component analysis. The screening of differential metabolites was performed using multivariate statistical analyses such as principal component analysis (PCA), orthogonal partial least squares‐discriminant analysis (OPLS‐DA), Student's *t* test, and the variable importance in the projection (VIP) of the first principal component of the OPLS‐DA model. Differential metabolites were annotated in the Fiehn Metabolome database based on retention time and mass‐to‐charge ratio (m/z). KEGG (Kanehisa, Goto, Furumichi, Tanabe, & Hirakawa, 2010) annotations were performed on the selected differential metabolites to retrieve all pathways for differential metabolite mapping. Thereafter, through a comprehensive analysis of the pathways of differential metabolites (including enrichment analysis and topological analysis), the pathways were further screened to identify the key pathways of the most relevant metabolite differences.

### 2.5 Characterization of metabolites

The identified metabolites were annotated using the Fiehn Metabolome database. The qualitative characterization of metabolites was compared with the Fiehn standard database based on their retention time. The degree of matching between the qualitative substance and the standard library substance was used as an indication of similarity. The possible perfect score was 1,000 points, with a score close to 1,000 indicated that the metabolite qualitative accuracy was higher.

## 3 RESULTS AND DISCUSSION

### 3.1 Typical total ion chromatogram of brown rice samples

**FIGURE 1:**
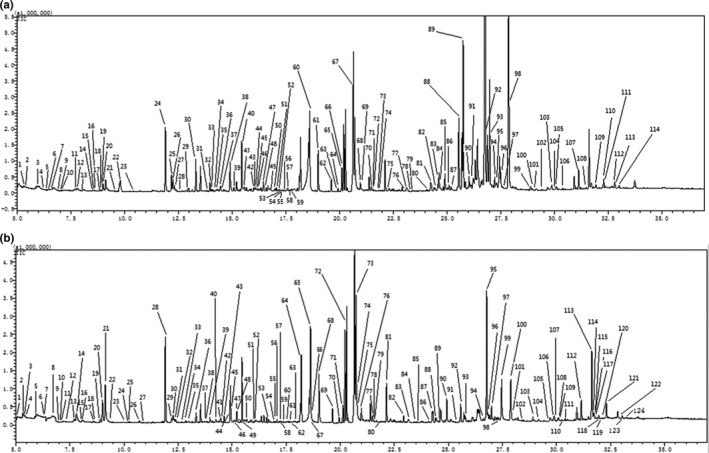
The total ion chromatogram of typical samples in the 15WC (a) and ZCWC (b). One hundred fifteen metabolites were identified in brown rice samples of 2015, and 122 metabolites were identified in stored samples. The baselines of the chromatographic peaks of the two groups of samples were stable, and the shapes of the chromatograms were roughly similar, but there were some differences, indicating that the metabolites of the brown rice samples changed before and after storage

A total of 297 peaks were detected in the 15WC brown rice samples, and 115 metabolites were identified. A total of 361 peaks were detected in the ZCWC samples, and 122 metabolites were identified. The total ion chromatograms of the two groups of samples are shown in Figure 1. It can be seen from this figure that the total ion chromatograms of the brown rice samples are roughly similar, but with certain differences, and the baseline of the peak is stable.

### 3.2 Qualitative results of metabolites in brown rice samples

The metabolites were characterized by the Fiehn database. The number of metabolites different between the two groups of brown rice samples was not much different, but the proportion of each type of substance differed. A total of 114 metabolites were identified in the control samples. In terms of quantity, the proportion of various substances is as follows: Sugars and their derivatives accounted for 26.32%; fatty acids and their derivatives accounted for 21.93%; amino acids and their derivatives accounted for 7.02%; alcohols and their derivatives accounted for 7.02%; phenolics, aldehydes, and acids accounted for 17.54%; sterols and glycerin accounted for 4.39%; and other classes such as amines, nitriles, alkanes, and purines accounted for 15.79%. On the other hand, the stored samples of 1 year were characterized by 124 metabolites. In terms of quantity, the proportion of various substances is as follows: Sugars and their derivatives accounted for 27.42%; fatty acids and their derivatives accounted for 20.97%; amino acids and their derivatives accounted for 8.06%; alcohols and their derivatives accounted for 7.26%; phenolics, aldehydes, and acids accounted for 16.94%; sterols and glycerin accounted for 4.84%; and other classes such as amines, nitriles, alkanes, and purines accounted for 14.52%. It can be seen from the total proportion of various types of substances that the proportion of fatty acids and their derivatives, and other substances decreased, while the content of amino acids, sugars and their derivatives, alcohols, sterols and glycerin, phenolics, aldehydes, and acids substances increased after storage. But the change in quantity ratio was small. A total of 160 metabolites were identified and analyzed qualitatively in both groups. The details of the metabolites are shown in Table 1. Among them, 114 metabolites were identified in brown rice samples in 2015, and 124 metabolites were identified in the stored samples, and 78 metabolites were common to both sample groups. In addition to the 78 metabolites found in both samples, the amino acid content of the metabolites specific to samples was different. The amino acids in the control samples were primarily threonine and proline, while the amino acid derivatives in the stored samples were citrulline, glutamic acid, trans‐4‐hydroxy‐L‐proline, and cysteine–glycine. These results indicate changes in the amino acid composition of brown rice during storage. Amino acids have a regulating effect on the growth of plants to ensure the normal physiological activities of the plants. Storage changes the environment of brown rice, such as cool and dark, and lower temperature. It may be that changes in the environment cause changes in amino acid composition. After storage, the amount of carbohydrates increases and turns into carbohydrate derivatives. Control samples contained lactulose, arabinose, levoglucosan, methyl O‐D‐galactopyranoside, etc., while the stored brown rice samples contained lactobionic acid, lactitol, arabitol, methylgalactose, 6‐deoxyglucose, etc. It means that the carbohydrates are metabolized. And sugars not only provide energy for brown rice metabolism, but also link up protein, lipid, nucleic acid metabolism, and secondary biomass metabolism. Changed in fatty acid species, from cinnamic acid, 2‐monostearin, gluconic acid lactone, oleic acid, linoleic acid methyl ester to monomyristin, 1‐monoolein, palmitic acid, 1‐monostearin, and arachidonic acid. After storage, alcohols, phenols, and acids also increased, indicating that fatty acids are decomposed and oxidized during storage to produce various intermediate products. Changes in the number and types of metabolites can indicate that the metabolism of brown rice has changed during storage, and the decrease in brown rice quality may be related to the number and types of metabolites.

**TABLE 1:** The qualitative results of metabolites

Number	Rt (min)	Quant mass	Metabolite name	Similarity	15WC	ZCWC
1	5.08	103	2‐hydroxybutanoic acid	424.3485	−	+
2	5.35	88	N‐methylalanine	718.0975	+	+
3	5.36	119	Hydroxylamine	833.9044	+	+
4	5.36	90	Unknown90	859.3294	+	−
5	5.96	119	o‐toluic acid	603.6677	+	+
6	6.30	117	Unknown117	776.5413	+	+
7	6.46	117	Capric acid	771.9091	+	+
8	6.53	119	Salicylaldehyde	793.3129	+	+
9	6.69	281	Unknown281	892.4862	+	+
10	7.45	281	Cerotinic acid	720.0099	+	+
11	7.71	120	Phenylalanine minor	653.1783	+	+
12	7.71	103	Lyxose	892.1435	+	+
13	7.80	97	Unknown97	885.2043	−	+
14	7.81	97	Isoleucine	921.1145	+	+
15	8.12	192	3‐(4‐hydroxyphenyl)propionic acid	583.8238	+	+
16	8.14	129	Benzoic acid	524.8588	+	+
17	8.48	299	Phosphate	875.6268	+	−
18	8.53	147	Glycerol	921.5075	+	+
19	8.57	136	2‐picolinic acid	805.9527	−	+
20	8.83	116	Adrenaline	652.1933	+	−
21	8.89	141	Cyanoalanine	865.6183	+	+
22	9.10	142	Proline	410.3996	+	−
23	9.13	155	Glutamine dehydrated 2	448.6608	−	+
24	9.30	150	2‐aminophenol	825.7078	−	+
25	9.50	103	Digitoxose	726.0803	+	+
26	9.77	148	Benzylalcohol	778.4053	+	−
27	9.80	148	Sulfuric acid	845.4259	−	+
28	10.38	163	Trans‐4‐hydroxy‐L‐proline minor1	484.9359	−	+
29	10.40	158	Leucine	735.8586	+	+
30	10.72	138	1‐methylgalactose	640.4558	−	+
31	10.87	144	Glutaric acid	400.8749	+	+
32	10.87	144	Citric acid	610.0528	−	+
33	11.33	86	Spermidine 3x	612.5784	+	+
34	11.73	191	Isochlorogenic acid	734.8947	+	+
35	11.86	239	Hydroquinone	490.6148	+	−
36	12.13	155	Thymidine	665.5627	+	+
37	12.25	129	Cholesterol	767.8018	+	+
38	12.32	103	2‐monoolein	763.2542	+	+
39	12.38	205	Cinnamic acid	565.6091	+	−
40	12.39	185	2‐piperidinobenzonitrile	497.7893	−	+
41	12.41	205	Hexadecylglycerol	534.4052	−	+
42	12.52	103	Fructose	643.0296	−	+
43	12.55	143	Phytol	697.1907	+	−
44	12.83	258	Uridine minor	652.1599	−	+
45	12.92	129	2‐monostearin	612.6996	+	−
46	12.97	184	Citrulline minor	529.3917	−	+
47	13.85	85	Tetracosane	374.1136	−	+
48	13.97	217	Deoxypentitol	755.0544	−	+
49	14.04	145	Butyraldehyde	479.6012	+	−
50	14.24	355	Epicatechin 1	706.168	+	+
51	14.25	355	Pseudouridine	687.2471	+	+
52	14.27	147	Pentono‐1,4‐lactone	724	+	+
53	14.35	129	2‐monopalmitin	715.4762	+	+
54	14.52	103	Lauric acid	654.7836	+	+
55	14.59	245	Fumaric acid	566.9534	+	+
56	14.91	97	Myristyl myristate	938.2184	+	+
57	15.00	145	Glutamic acid	630.4272	−	+
58	15.09	277	Indoxyl sulfate	589.7618	−	+
59	15.12	129	3‐deoxyerythritol	564.3019	+	+
60	15.21	204	Levoglucosan	939.8019	+	−
61	15.27	85	Arabitol	646.8239	−	+
62	15.28	145	Xylitol	670.5211	−	+
63	15.56	87	Unknown87	800.8511	+	−
64	16.01	129	N‐acetyl‐D‐mannosamine minor1	630.1867	+	−
65	16.09	217	glucose‐1‐phosphate	932.3543	+	+
66	16.09	271	2,4‐hexadienedioic acid	648.5786	+	−
67	16.12	271	sucrose‐6‐phosphate	773.9161	−	+
68	16.23	145	Fructose 2	606.5877	+	−
69	16.26	116	Pentonic acid	753.9878	+	+
70	16.59	116	Gluconic acid lactone	680.368	+	−
71	16.60	116	Palatinitol	808.9568	+	+
72	16.60	85	1,5‐anhydroglucitol	805.4726	+	+
73	16.63	85	Sorbitol	561.0001	−	+
74	16.91	87	Pinitol	813.12	+	−
75	17.01	103	Ribose	873.2284	+	+
76	17.06	119	Glycerol‐3‐galactoside	439.3125	+	−
77	17.10	159	Ononitol	662.7111	−	+
78	17.20	237	Unknown237	797.7854	+	−
79	17.23	147	Myristic acid	742.7762	+	+
80	17.42	103	Tagatose 2	915.0256	+	+
81	17.47	103	Arabinose	725.1438	+	−
82	17.55	204	Methylhexose nist	764.2579	+	+
83	17.63	263	Methyl O‐D‐galactopyranoside	622.6544	+	−
84	17.73	318	Conduritol‐beta‐epoxide minor	703.9177	+	+
85	18.13	87	N‐acetyl‐D‐mannosamine minor2	719.4688	−	+
86	18.49	204	Glucose non‐meox	789.4738	+	+
87	18.50	205	Galactose‐6‐phosphate 2	726.5164	−	+
88	18.59	217	UDP‐glucuronic acid	921.2144	−	+
89	18.59	149	Myo‐inositol	722.2712	−	+
90	19.04	237	Alpha tocopherol	671.4877	+	+
91	19.63	117	Palmitic acid	964.5748	+	+
92	19.93	355	Epigallocatechin	693.4798	+	−
93	19.95	204	N‐acetylgalactosamine	899.6354	+	+
94	20.11	305	Conduritol‐beta‐epoxide	898.4097	+	+
95	20.29	97	Hypoxanthine	596.1447	+	+
96	20.71	97	d6 cholesterol	769.4017	+	+
97	20.80	105	Arachidonic acid	584.2689	−	+
98	20.84	241	Cysteine‐glycine minor2	500.2469	−	+
99	20.92	204	Glycerol‐3‐galactoside 2	469.9341	−	+
100	21.12	204	Lactulose 1	655.02	+	−
101	21.22	105	Hippuric acid 2	584.8744	+	+
102	21.51	138	Linoleic acid	852.2582	+	+
103	21.58	314	Unknown314	723.1386	+	+
104	21.59	117	Oleic acid	889.0658	+	−
105	21.86	117	Stearic acid	836.2811	+	+
106	21.89	93	Linoleic acid methyl ester	540.8296	+	−
107	22.36	89	Linolenic acid	738.458	+	+
108	22.53	97	Cis‐gondoic acid	775.2384	+	+
109	23.01	99	Glucoheptulose	732.4016	+	+
110	23.42	129	N‐acetylmannosamine	663.4083	+	−
111	23.45	343	Monomyristin	717.6167	−	+
112	24.46	299	Ethanolamine	544.2243	+	−
113	24.48	117	Palmitic acid	745.136	−	+
114	24.49	319	Sophorose minor1	851.0139	−	+
115	24.50	204	Lactobionic acid	780.4049	+	+
116	24.66	361	1‐kestose	901.8181	+	+
117	24.73	319	Glucose overload	656.6479	+	+
118	24.91	89	Octadecanol	596.2013	−	+
119	25.22	361	Diglycerol	498.0914	+	−
120	25.34	371	1‐monopalmitin	795.2885	+	+
121	25.56	217	4′,5‐dihydroxy‐7‐glucosyloxyflavanone degr1	582.5474	+	−
122	25.75	361	Sucrose	942.2086	+	+
123	25.94	131	2‐hydroxybutanoic acid	399.0007	+	−
124	26.16	98	Uridine	648.1599	−	+
125	26.24	289	Threonine minor	511.3119	+	−
126	26.78	98	1‐monoolein	735.6812	−	+
127	26.82	89	2‐ketoisovaleric acid	470.2351	+	−
128	27.10	399	1‐monostearin	798.8755	−	+
129	27.37	127	Coniferin	669.9934	+	+
130	27.43	89	3,6‐anhydro‐D‐glucose minor2	729.3417	+	−
131	27.45	95	squalene	909.123	+	+
132	27.51	204	Cellobiose minor	702.9962	+	−
133	27.62	117	Lignoceric acid	492.3313	+	+
134	27.83	204	Maltitol	841.4262	+	+
135	27.87	361	Lactitol	659.9863	−	+
136	27.94	204	Lactobionic acid	803.1534	−	+
137	28.49	87	Unknown88	744.8497	−	+
138	28.83	204	Galactinol	879.7164	+	+
139	28.95	135	Tocopherol gamma‐	707.3479	+	+
140	29.17	89	Fructose‐1‐phosphate	718.9215	−	+
141	29.45	151	Maltotriose 1	741.5077	+	+
142	29.52	204	Beta‐mannosylglycerate	864.7857	+	+
143	29.69	117	Piceatannol 3	679.285	+	+
144	29.75	89	Formononetin	724.0859	−	+
145	30.09	165	Tocopherol acetate	833.6448	+	−
146	30.11	237	Tocopherol alpha‐	869.78	+	+
147	30.25	117	6‐deoxyglucose	544.1461	−	+
148	30.40	151	Phenol	316.6205	+	+
149	31.23	129	Stigmasterol	859.5956	+	+
150	31.68	129	Beta‐sitosterol	814.7219	+	+
151	31.68	495	Triacontanol	482.7464	−	+
152	31.75	215	Dihydrocholesterol	615.7613	−	+
153	31.96	361	Inulotriose 2	900.1816	+	+
154	32.20	441	Unknown441	839.3766	+	+
155	32.30	95	Lanosterol	690.7017	+	+
156	32.73	124	Digalacturonic acid	652.1204	+	−
157	32.82	95	Lithocholic acid	653.1404	+	+
158	33.03	136	Chenodeoxycholic acid	605.6545	+	+
159	33.09	136	Cellobiose minor	567.6136	−	+
160	34.31	217	Melezitose	814.7955	+	+

Note

Rt stands for retention time; Quant mass represents the mass‐to‐core ratio; Metabolite name represents the metabolite name; Similarity is the degree to which the qualitative substance matches the substance in the Fiehn standard library; 15WC represents the sample of the 2015 Wuchang area; ZCWC represents a sample of the Wuchang area after storage; “+” means that it contains this metabolite; and “−” indicates that metabolites could not be detected because the content of metabolites is very low or has fallen below the machine's sensitivity threshold.

### 3.3 Multivariate statistical analysis of brown rice metabolites

**FIGURE 2:**
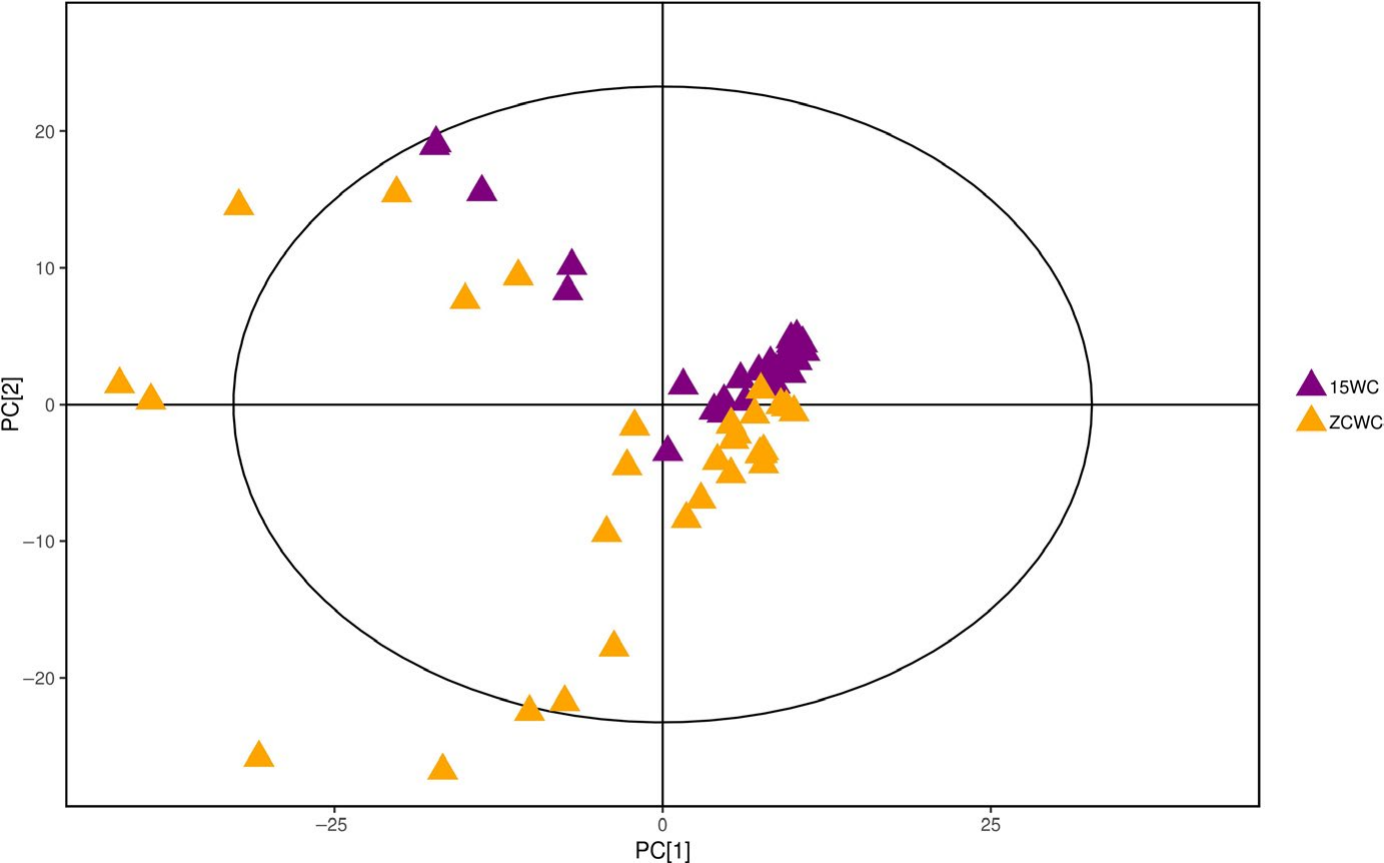
The score scatter plot of PCA model for group 15WC versus ZCWC. The brown rice sample of 2015 and the stored brown rice sample were basically in the 95% confidence interval. The two groups of samples had clustered areas with similar distribution patterns. The two groups of samples could be better distinguished, indicating that the metabolism of brown rice samples was different due to storage. The larger the distance between sample points after storage, the larger the difference between the samples

Multivariate statistical analysis was performed for the control and stored brown rice samples to show the differences in their metabolites. The PCA reflects the original state of the metabolomic data and can explain the variables. Figure 2 is a scatter plot of the PCA model of two sets of brown rice samples. The 15WC samples and the ZCWC samples were mainly within the 95% confidence interval. The two groups of samples had corresponding clustering regions with similar distribution patterns. The two groups had a small overlapping portion and, therefore, were not completely distinguished. This indicates that storage caused a difference in the metabolism of the brown rice samples. The larger distance between the sampling points poststorage indicates that storage affected the metabolites content, making the differences between the samples larger. A total of five principal components were obtained in this analysis, with cumulative *R* ^2^ *X* = 0.517, *Q* ^2^ = 0.35, and *Q* ^2^ < 0.5, indicating that the model was not very effective.

**FIGURE 3:**
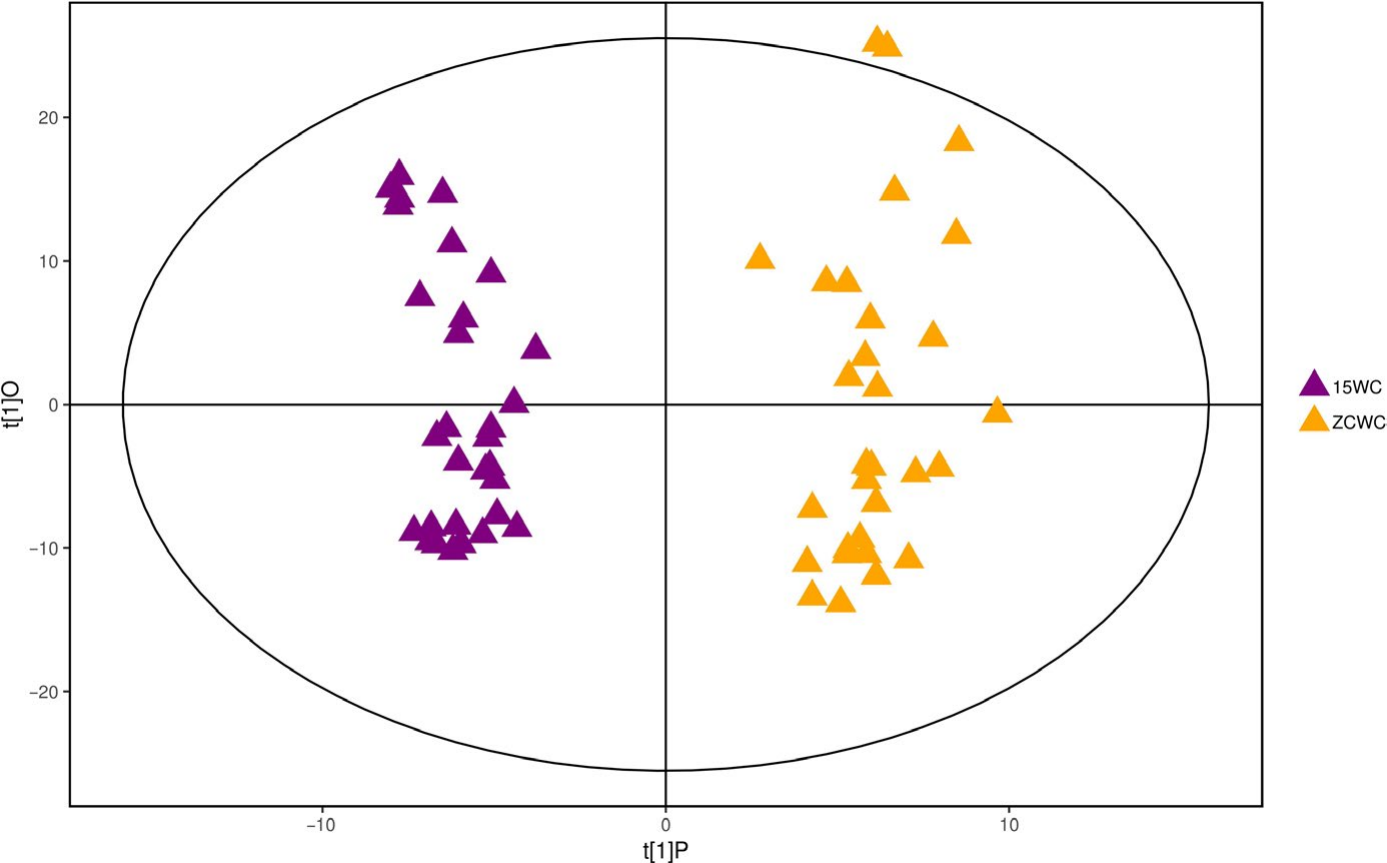
The score scatter plot of OPLS‐DA model for group 15WC versus ZCWC. Control samples and stored samples were located on the left and right sides of the confidence interval. The two groups of samples were clearly distinguished, and their distribution patterns were quite different. There is a certain distance between the control samples, indicating that there are also metabolic differences within each group of samples. Compared with the control sample, the distance between the stored samples is larger, which indicates that the storage has changed the metabolites of the brown rice samples and increased the difference

Principal component analysis will disperse the difference variables to more principal components, which cannot be better visualized and subsequently analyzed. OPLS‐DA can filter out orthogonal variables that are not related to categorical variables in metabolites, thereby obtaining more reliable differences between groups of metabolites. As shown in Figure 3, the two groups of brown rice samples are mainly found within the 95% confidence interval, and the two groups of samples are significantly different. The 15WC samples are located on the left side of the confidence interval, while the ZCWC samples are found on the right side of the confidence interval. The distinction worked well. Based on the distribution pattern, the distance between the samples in the ZCWC group was significantly larger than the distance between 15WC samples. This indicates that storage changed the metabolites of brown rice samples and increased the differences between the groups. There was also a certain distance between the 15WC samples, indicating that there were also metabolic differences between the samples, which may be related to the different locations from where the samples were collected. This analysis has two main components; *R* ^2^ *X* = 0.322, *R* ^2^ *Y* = 0.958, *Q* ^2^ = 0.932, and the *Q* ^2^ value was close to 1. This result indicates that the OPLS‐DA model has good predictability and no overfitting phenomenon. It can be seen from the OPLS‐DA score map that the storage period has a significant effect on brown rice metabolism.

**FIGURE 4:**
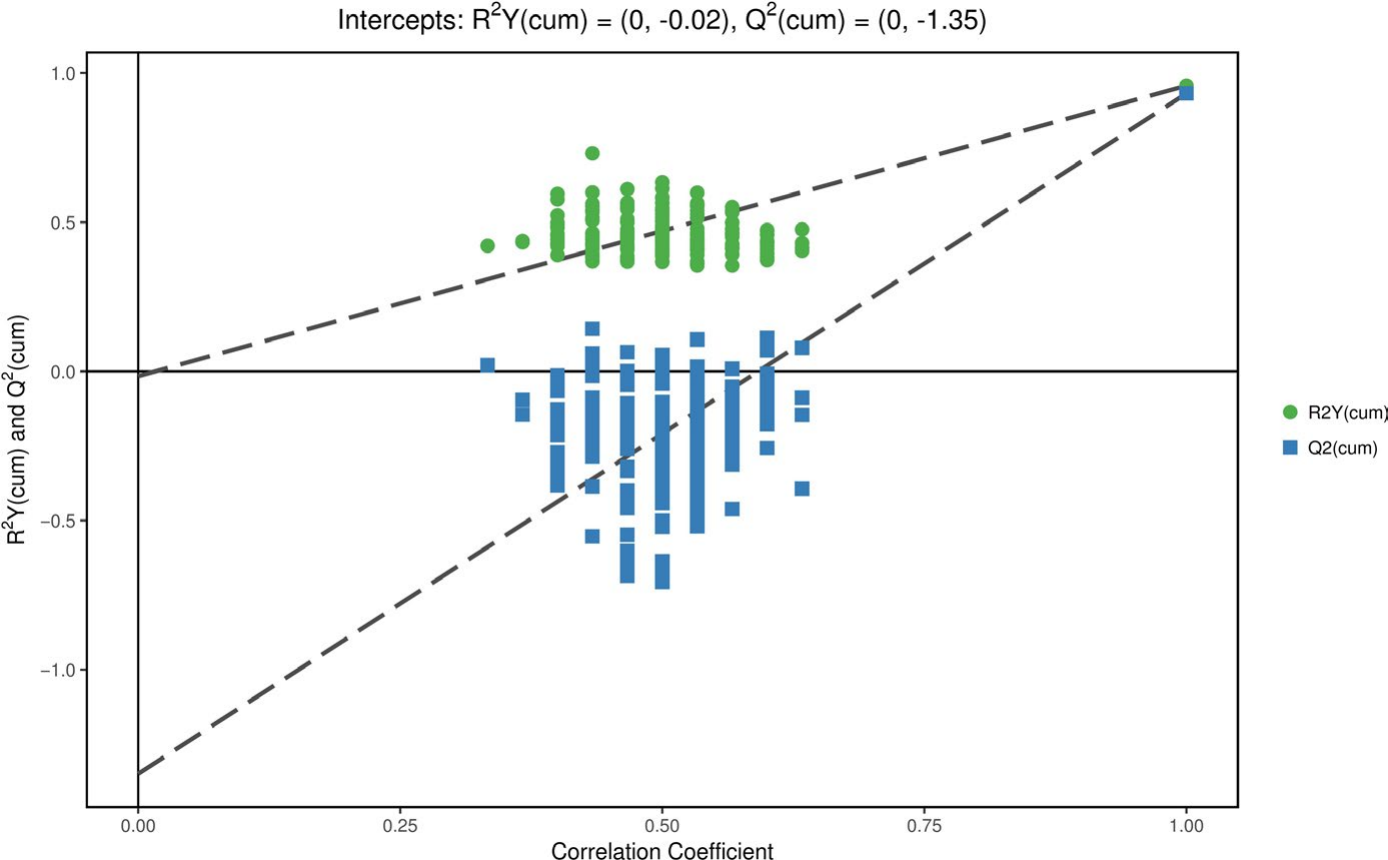
The permutation test of OPLS‐DA model for group 15WC versus ZCWC. The abscissa represents the permutation retention of the permutation test, and the ordinate indicates the value of *R* ^2^ *Y* or *Q* ^2^. The green dot indicates the *R* ^2^ *Y* value obtained by the permutation test, the blue square indicates the *Q* ^2^ value obtained by the permutation test, and the two dotted lines indicate the regression lines of *R* ^2^ *Y* and *Q* ^2^, respectively. The original model *R* ^2^ *Y* is very close to 1, indicating that the established model conforms to the real situation of the sample data. The original model *Q* ^2^ is very close to 1, indicating that if a new sample is added to the model, an approximate distribution will be obtained. In general, the original model can be used to explain the difference between the two groups of samples. The *Q* ^2^ value of the random model of permutation test is smaller than the *Q* ^2^ value of the original model, the intercept of the regression line of *Q* ^2^ and the vertical axis is <0, the proportion of the *Y* variable increases with the decrease in permutation retention, and the *Q* ^2^ of the stochastic model gradually decreases. This shows that the OPLS‐DA model has good robustness and there is no overfitting phenomenon

The permutation test establishes the corresponding OPLS‐DA model by randomly changing the order of the categorical variable *Y* (multiple times *n* = 200) to obtain the *R* ^2^ *Y* and *Q* ^2^ values of the stochastic model. It plays an important role in avoiding the overfitting of the test model and evaluating the statistical significance of the model. The results of the permutation test of the OPLS‐DA model are shown in Figure 4.

In Figure 4, the abscissa represents the permutation retention of the permutation test, and the ordinate indicates the value of *R* ^2^ *Y* or *Q* ^2^. The green dot indicates the *R* ^2^ *Y* value obtained by the permutation test, the blue square indicates the *Q* ^2^ value obtained by the permutation test, and the two dotted lines indicate the regression lines of *R* ^2^ *Y* and *Q* ^2^, respectively. The *R* ^2^ *Y* value of the original model was very close to 1, indicating that the established model conforms to the real situation of the sample data.

The *Q* ^2^ value of the original model was very close to 1, indicating that if a new sample is added to the model, an approximate distribution will be obtained. In general, the original model can be used to explain the difference between the two groups of samples. The *Q* ^2^ value of the random model of permutation test was smaller than the *Q* ^2^ value of the original model, the intercept of the regression line of *Q* ^2^ and the vertical axis was <0, the proportion of the *Y* variable increased with the decrease in permutation retention, and the *Q* ^2^ of the stochastic model gradually decreased. This shows that the original model has good robustness and there is no overfitting phenomenon.

### 3.4 Screening and analysis of differential metabolites with significant changes in content

“Differential metabolites” refers to substances which were found in both samples but showed significant differences in content. The VIP (variable importance in the projection) value of the OPLS‐DA model (threshold ≥1) was combined with the *p* value of the Student's *t* test (threshold <0.05) to search for differential metabolites. In the Fiehn database, the substances in the library were matched by the retention time and other conditions, and the qualitative metabolites were characterized. The qualitative results of the differential metabolites are shown in Table 2.

**TABLE 2:** Differences in qualitative results of metabolites between 15WC and ZCWC samples

	Metabolite name	Similarity	Rt	Mass	VIP	*p*‐VALUE	FOLD CHANGE	LOG_FOLDCHANGE
1	Isoleucine	937	5.26	126	1.583	0.018449226	2.0928	+1.0655
2	o‐toluic acid	844	5.27	119	1.917	1.59915E‐05	2.4738	+1.3067
3	N‐methylalanine	718	5.35	88	1.899	8.38585E‐05	2.8456	+1.5087
4	Unknown117	776	6.3	117	1.838	0.037261411	0.6793	−0.5580
5	capric acid	820	6.47	117	2.199	9.36425E‐05	0.4344	−1.2028
6	Unknown218	892	6.69	281	1.501	0.000796171	1.7564	+0.8127
7	lyxose minor	892	7.71	103	1.255	8.01264E‐05	0.3279	−1.6085
8	3‐(4‐hydroxyphenyl)propionic acid	565	8.11	192	3.430	2.34821E‐10	91,883.9835	+16.4875
9	glutaric acid	400	10.87	144	3.303	1.13885E‐10	27.6803	+4.7908
10	spermidine 3x	612	11.33	86	1.419	5.08101E‐05	3.1033	+1.6338
11	isochlorogenic acid	734	11.73	191	3.120	6.70567E‐13	5.2718	+2.3983
12	Thymidine	763	12.11	88	1.580	0.001239873	2.4768	+1.3085
13	Deoxypentitol	764	13.98	217	1.046	0.001006206	0.6278	−0.6715
14	pseudouridine	687	14.25	355	1.183	0.022955145	0.6442	−0.6344
15	pentono‐1,4‐lactone	724	14.27	147	1.932	0.000180756	0.4964	−1.0105
16	lauric acid	654	14.52	103	3.114	2.13247E‐10	0.0887	−3.4942
17	3‐deoxyhexitol	564	15.12	129	2.758	4.00841E‐10	0.4916	−1.0245
18	Palatinitol	808	16.6	116	1.682	2.81239E‐06	0.3898	−1.3593
19	galactinol 4	933	18.12	204	1.052	0.017682323	0.4707	−1.0872
20	glucose non‐meox	789	18.49	204	1.700	0.000458856	2.5573	+1.3546
21	beta‐mannosylglycerate	896	21.17	204	1.219	5.28029E‐10	0.2106	−2.2472
22	hippuric acid 2	598	21.23	105	1.274	0.000147006	2.1331	+1.0930
23	Unknown314	723	21.58	314	1.554	0.004029503	1.9027	+0.9280
24	cis‐gondoic acid	775	22.53	97	1.287	0.044331513	1.4018	+0.4873
25	glucoheptulose	782	23.13	126	1.693	3.2738E‐07	3.9232	+1.9720
26	2‐monopalmitin	872	25	511	2.629	3.58945E‐07	5.5777	+2.4797
27	diglycerol	631	25.23	244	1.367	0.022536718	0.8052	−0.3126
28	coniferin	748	26.38	98	1.316	0.020529403	1.5673	+0.6483
29	lignoceric acid	507	27.63	117	2.040	1.94613E‐08	2.1027	+1.0723
30	lithocholic acid	731	30.51	95	1.278	0.003767164	0.6629	−0.5931
31	Unknown441	839	32.16	441	2.339	5.52911E‐06	0.2403	−2.0572

Note

Metabolite name, the name of the substance in the Fiehn database; Similarity: The substance matches the peak of the mass spectrometric detection; VIP: the VIP value from the OPLS‐DA model; Mass: the mass‐to‐charge ratio of the substance of the substance; *p*‐value: The *p*‐value from Student's t test; FOLD_CHANGE: the ratio of the two groups of experimental substances; LOG_FOLDCHANGE: FOLD CHANGE takes the base 2 logarithm; positive sign indicates that 15WC increases the relative content of ZCWC; and negative sign indicates that 15WC decreases the relative content of ZCWC.

The two sample groups 15WC and ZCWC were compared. A total of 31 differential metabolites were screened, of which four were unknown. As can be seen in Table 2, the content of 17 metabolites decreased after storage (including fatty acids, amino acids, sugars, organic acids, alcohols, and amines), and 14 metabolites were elevated (including fatty acids, sugars, and polyglycosides). The reduced amino acid content may be due to decomposition during storage. After being decomposed into α‐ketocarboxylic acids and amines, they may be converted into sugars or lipids, or some nonessential amino acids may be synthesized. The carbon framework of amino acids is α‐keto acid, which is an intermediate product of sugar metabolism. Sugars and lipids can be converted to each other because glycerin can be reversed into hexose, and fatty acids may be converted into sugar after decomposing into acetyl‐CoA. Therefore, the substances with increased and decreased content in differential metabolites undergo metabolic transformation during storage, resulting in significant changes in content. The quality of brown rice changes after storage, and the change in various metabolite contents may be related to the change of brown rice quality.

### 3.5 Analysis of trends in differential metabolite content

**FIGURE 5:**
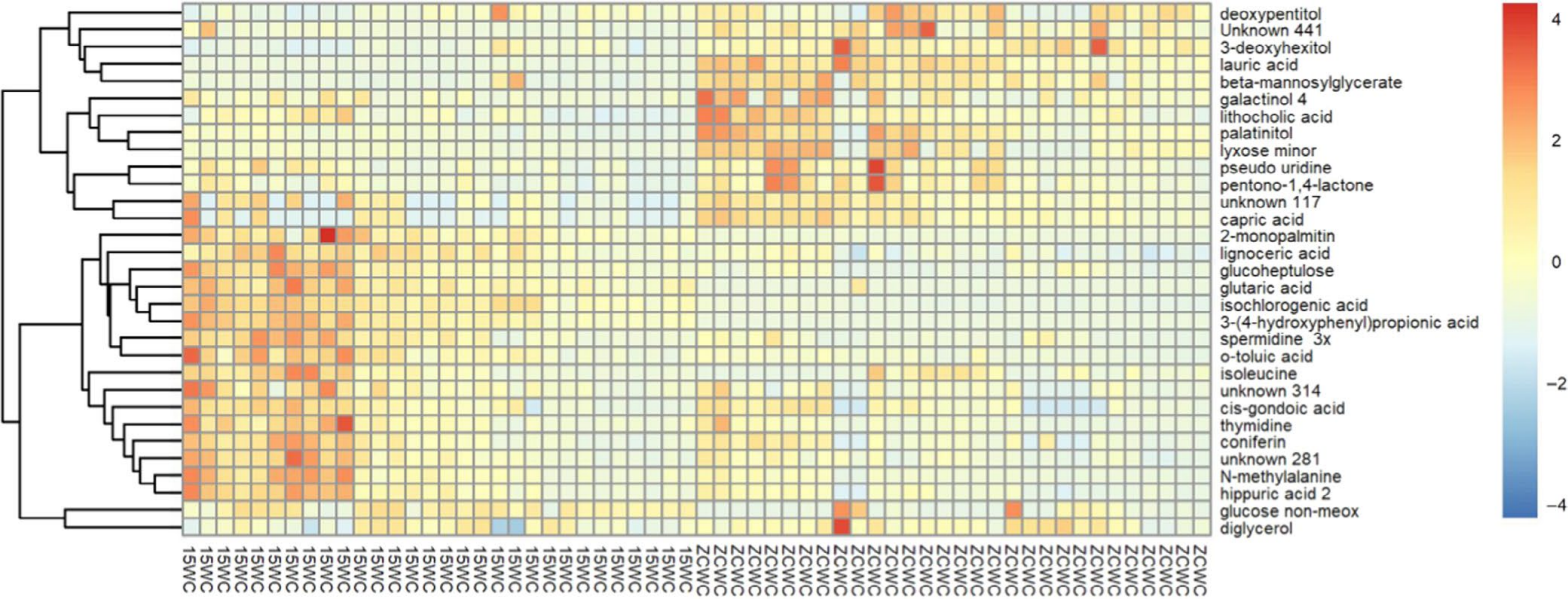
The heatmap of hierarchical clustering analysis for group 15WC versus ZCWC. The abscissa represents different experimental groups. The left side shows the 15WC samples, and the right part represents the ZCWC samples. The ordinate represents the differential metabolites of the group. The color blocks at different positions represent the relative expression of metabolites in the corresponding position. The red blocks represent high expression levels, and the blue blocks represent low expression levels. Cluster analysis found that the lowly expressed metabolites in the 2015 samples became highly expressed after storage, while highly expressed metabolites in 2015 samples became lowly expressed after storage. Storage caused a “reverse change” in the metabolites content of brown rice

The differential metabolites obtained by the above analysis often have structural and functional similarities/complementarities or are positively/negatively regulated by the same metabolic pathway, showing similar or opposing expression characteristics between different experimental groups. Hierarchical clustering analysis of these characteristic features aids in classification of metabolites with similar characteristics into one class, and determination of variations in the characteristics of metabolites between experimental groups. The Euclidean distance matrix was calculated for the quantitative values of the differential metabolites, and the clustering of differential metabolites was carried out using a complete linkage method and displayed by a thermogram. The results are shown in Figure 5.

As can be seen in the figure, the abscissa represents different experimental groups. The left side shows the 15WC samples, and the right part represents the ZCWC samples. The ordinate represents the differential metabolites of the group. The color blocks at different positions represent the relative expression of metabolites in the corresponding position. The red blocks represent high expression levels, and the blue blocks represent low expression levels. Figure 5 can be divided into upper and lower areas. The first 14 metabolites on the right side of the figure are the upper area, and the color difference between the left and right sides is significant. It can be seen that deoxypentitol, Unknown441, 3‐deoxyhexitol, galactinol 4, lithocholic acid, lyxose minor, palatitol, pentono‐1,4‐lactone, pseudouridine, capric acid, beta‐mannosylglycerate, Unknown117, and lauric acid levels in the ZCWC samples are significantly higher than in the 15WC samples, The reason may be that the oxidation and hydrolysis of lipids, the enzymatic hydrolysis of starch, and the metabolism of sugar all lead to an increase in the content of these metabolites. Unknown441 is located between deoxypentitol and 3‐deoxyhexitol, indicating that the three components are metabolites with the same characteristics; if so, Unknown441 may be a polyol. Unknown117 is located between pentono‐1, 4‐lactone and lauric acid, indicating that this metabolite may be a fatty acid or a fatty acid derivative. The other 17 differential metabolites are in the lower region. As indicated by their color, the content of these 17 differential metabolites was lower in the stored samples than in the 2015 brown rice sample, indicating that the content of these substances decreased after storage. Most of these 17 metabolites were fatty acids, while a small number were organic acids, sugars, and amino acids. The content of some fatty acids increases after storage, and some of them decreased, indicating that the change in fatty acid content is a dynamic process. Changes in fatty acid content after storage were more pronounced than other metabolites. The metabolite Unknown314 was located between isoleucine and cis‐gondoic acid, indicating that it may be a fatty acid. Unknown281 was located between coniferin and N‐methylalanine. This metabolite may therefore be a derivative of amino acids or a glycoside. In Figure 5, comparing the 15WC sample on the left side with the ZCWC on the right side, it can be seen that the high‐content metabolites in the 15WC sample were low after storage, while the low‐level metabolites become high after storage. Storage changed the content of the metabolites of the brown rice in a “reverse change,” that is, metabolites with high content became low content while metabolites with low content turned high.

### 3.6 Effect of storage on brown rice metabolic pathways

The 31 differential metabolites were analyzed by KEGG annotation to find all pathways involved in regulating these differential metabolites. The key pathways most relevant to metabolite differences, which are the biosynthesis pathway of fatty acids, were found by comprehensive analysis of pathways (including enrichment analysis and topological analysis). This result indicates that storage has the greatest effect on fatty acids in brown rice. The results of this experiment indicate that lauric acid is a key metabolite in the fatty acid synthesis pathway in Wuchang brown rice samples. Lauric acid is a free fatty acid, and its content of free fatty acids increases after storage. A previous study investigated lipid changes in brown rice after storage in a controlled atmosphere and found that the free fatty acid content increased with storage time. Xu et al. (2005) studied the quality changes of Wuchang brown rice during storage. The fatty acid value of brown rice in the accelerated aging experiment using high temperature and high humidity was measured. It was found that the fatty acid content of Wuchang brown rice increased with storage time. Other studies have also confirmed the structural changes and changes in fatty acid content during storage. The present study investigated metabolite changes after storage of Wuchang brown rice from the perspective of metabolomics. Compared with the results of total fatty acid values determined in the above studies, the trend was the same. Rice aging is a very complex process involving changes in lipids, starches, proteins, and enzymes, but lipid changes are currently considered to be the most important cause of rice aging. In this study, we studied the change in fatty acid content from the perspective of metabolite content before and after storage. It was found that the change in fatty acid content was significant in all metabolites. The fatty acids belonged to the lipid class, so metabolomics can also explain that storage has the greatest impact on lipids in brown rice, and can be accurate to specific lipid metabolites.

## 4 CONCLUSIONS

Based on the metabolomics technology of GC‐MS, a metabolomics analysis was performed on stored brown rice. The results showed that storage had a small effect on the amount of brown rice metabolites and a large effect on the composition of metabolites. In terms of content, storage had a significant effect on lipids and sugars. The content showed a “reverse change” trend. Storage had the greatest effect on fatty acid synthesis pathways. Lauric acid was a key metabolite for lipid metabolism during brown rice storage. The degradation of brown rice quality caused by storage was closely related to the composition and content of its metabolites. Lipids were the main factors affecting brown rice quality during storage.

## CONFLICT OF INTEREST

The authors declare that they have no competing interests.

## ETHICAL APPROVAL

This article does not contain any studies with human participants or animals performed by any of the authors.

## INFORMED CONSENT

Informed consent was obtained from all individual participants included in the study.
